# ILOBONE: A phase I/IIa randomized controlled trial to assess the safety and feasibility of local iloprost therapy for enhancing proximal humerus fracture healing– a pilot study design

**DOI:** 10.1186/s13018-025-05865-2

**Published:** 2025-05-22

**Authors:** Hisham Elazaly, Ioanna Maria Dimitriou, Tazio Maleitzke, Michael Dahne, Vera Jaecker, Sven Maerdian, Sascha Tafelski, Torsten Diekhoff, Tobias Lindner, Doruk Akgün, Anna-Maria Mielke, Alp Paksoy, Dominik Adl Amini, Elisa Marie Planatscher, Vincent Leopold, Susana González-Khatib, Paul Christoph Köhli, Marcel Niemann, Alexander Hildebrandt, Stephan Oehme, Yannick Palmowski, Melissa Paraskevaidis, Lukas Schönnagel, Sebastian Benedict Braun, Matthias Pumberger, Sebastian Hardt, Sigmar Stricker, Levent Akyüz, Gerald Grütz, Stefan Schaller, Luis Lauterbach, Maximilian Volcksdorff, Lukas Mödl, Martin Textor, Melanie Ort, Simon Reinke, Ulrich Stöckle, Carsten Perka, Georg N. Duda, Katharina Schmidt-Bleek, Sven Geissler, Tobias Winkler

**Affiliations:** 1 Center for Musculoskeletal Surgery, Charité– Universitätsmedizin Berlin, Corporate Member of Freie Universität Berlin and Humboldt-Universität zu Berlin, Berlin, Germany; 2 Berlin Institute of Health (BIH) at Charité– Universitätsmedizin Berlin, Julius Wolff Institute (JWI), Berlin, Germany; 3 Berlin Institute of Health (BIH) at Charité– Universitätsmedizin Berlin, BIH Center for Regenerative Therapies (BCRT), Berlin, Germany; 4 Department of Biology, Chemistry and Pharmacy, Institute of Chemistry and Biochemistry, Freie Universität Berlin, Berlin, Germany; 5 Berlin Institute of Health (BIH) at Charité– Universitätsmedizin Berlin, BIH Biomedical Innovation Academy, BIH Charité Clinician Scientist Program, Berlin, Germany; 6 Department of Orthopedic Surgery, Copenhagen University Hospital- Amager and Hvidovre, Hvidovre, Denmark; 7 Department of Clinical Medicine, University of Copenhagen, Copenhagen, Denmark; 8 Department of Radiology, Charité– Universitätsmedizin Berlin, Corporate Member of Freie Universität Berlin and Humboldt-Universität zu Berlin, Berlin, Germany; 9 Emergency Department, Charité– Universitätsmedizin Berlin, Corporate Member of Freie Universität Berlin and Humboldt-Universität zu Berlin, Berlin, Germany; 10 Berlin Institute of Health (BIH) at Charité– Universitätsmedizin Berlin, BIH Biomedical Innovation Academy, BIH Charité Junior Clinician Scientist Program, Berlin, Germany; 11 Institute of Biometry and Clinical Epidemiology, Charité - Universitätsmedizin Berlin, Corporate Member of Freie Universität Berlin and Humboldt-Universität zu Berlin, Berlin, Germany; 12 Berlin Center for Advanced Therapies (BECAT), Charité– Universitätsmedizin Berlin, Corporate Member of Freie Universität Berlin and Humboldt-Universität zu Berlin, Berlin, Germany

**Keywords:** Proximal humerus fracture, Bone healing, Fracture repair, Iloprost, Ilomedin, PHILOS

## Abstract

**Background:**

Proximal humerus fractures (PHFs) are the third most common fractures in elderly patients. Over 70% of PHFs in patients aged over 60 are displaced fractures, often necessitating surgical treatment. However, osteosynthesis is associated with a high rate of complications, highlighting the urgent need for additional therapeutic approaches to enhance bone healing and prevent osteonecrosis. This study evaluates the safety, feasibility and potential efficacy of local prostacyclin (iloprost) to improve bone healing in patients with PHFs.

**Methods:**

Thirty eligible patients will be randomized into one of three groups at a 1:1:1 ratio. All patients will receive angular stable locking plate fixation. Two treatment groups will receive an additional single dose of local iloprost through a 24-hour infusion postoperatively (group 1: low dose; group 2: high dose), while the control group will only receive the osteosynthesis. Patients will be monitored for 52 weeks. The primary endpoint is safety, with secondary endpoints including the preservation of the screw tip apex distance as an indicator of fracture healing, head shaft angle, necrosis rate, and patient-related outcome measures.

**Discussion:**

The Ilobone study aims to provide data on the potential for biological augmentation of osteosynthesis procedures in PHFs, prone to healing challenges and complications.

**Trial registration:**

The trial is registered with ClinicalTrial.gov (NCT04543682), registered 02 Sep. 2020, https://clinicaltrials.gov/show/NCT04543682 and the German Clinical Trials Registry (DRKS00027081), registered 10 Nov. 2021 https://drks.de/search/de/trial/DRKS00027081.

**Supplementary Information:**

The online version contains supplementary material available at 10.1186/s13018-025-05865-2.

## Background

Proximal humerus fractures (PHFs) are the third most common fractures in patients aged 65 and older, following hip and distal radius fractures [1]. The incidence of PHF is expected to triple over the next three decades due to the progressive aging of the world’s population [2]. The majority of PHFs are nondisplaced and can be treated conservatively, yielding satisfactory results [3]. However, Neer [4] type III and IV fractures are often treated surgically, as conservative treatment leads to unsatisfactory results with worse functional outcomes and a reduced quality of life [3, 5]. More than 70% of PHFs in patients over 60 years of age are displaced fractures requiring surgical intervention [6]. Open reduction and internal fixation is associated with a high rate of complications (up to 57%) in elderly patients, including screw cut-out, malunion, nonunion, avascular necrosis, and infection [6, 7].

Fracture healing is a complex process involving several sequential and overlapping phases. The first phase includes an initial inflammatory response characterized by activating monocytes and macrophages and accumulating adaptive immune cells such as CD8 + T cells or B cells [8, 9]. This phase is associated with locally increased levels of inflammatory cytokines such as interleukin-6 (IL-6), interferon-gamma (IFNɣ) and tumor necrosis factor-alpha (TNFα) [10, 11]. The timely resolution of this acute inflammation is essential for the subsequent phases of the bone healing cascade, which restores the original architecture of the injured tissue [12, 13]. The transition from the initial proinflammatory to an anti-inflammatory environment is synchronized with an increased release of angiogenic and osteogenic growth factors [13].

Consequently, prolonging this proinflammatory response impairs angiogenesis and osteogenesis, resulting in delayed healing or nonunions [13]. The negative effect of excessive proinflammatory activity on bone healing has been extensively described in patients with autoimmune diseases or cancer [14]. These individuals often suffer from impaired bone regeneration, which is linked to a greater number of immune cells and elevated levels of proinflammatory cytokines in their fracture hematomas and surrounding bone marrow compared to those in “healthy” fracture patients [14].

Recent studies have also demonstrated the high relevance of the initial inflammatory response in successfully initiating bone healing in “normal” fracture patients and have specifically identified T cells as essential modulators of the healing process. In particular, terminally differentiated effector memory CD8 + T cells (CD8 + T_EMRA_) have been significantly correlated with poor fracture healing [15, 16]. Their numbers increase with age and correlate with chronic antigen exposure and general immune system experience [15]. CD8 + T_EMRA_ cells can be activated and exert strong effector functions, such as cytotoxicity and cytokine release, without the need for T-cell receptor cross-linking by antigen exposure. This is due to bystander activation via cytokine receptors [15]. CD8 + T_EMRA_ cells, which are locally the major producers of TNF-α and IFN-γ, have been found enriched in the hematomas of patients with impaired healing. Consequently, they prolong the proinflammatory phase and prevent its timely resolution, affecting subsequent healing steps. The prolonged proinflammatory phase limits pro-osteogenic cell function, reducing osteogenesis in mice and humans [15, 16].

However, regulatory CD4 + T cells (CD4 + T_reg_), considered mainly counterparts to effector T cells, are also involved in regulating the inflammatory response and promoting angiogenesis, with beneficial effects on wound and bone healing [16, 17]. Therefore, the balance between regulatory and effector memory T cells is critical, as both a prolonged or absent inflammatory response can lead to tissue damage and delayed healing. These data indicate that a balanced immune cascade is essential for adapting the local fracture environment to the appropriate healing phase and promoting bone healing [11, 13].

In this study, iloprost will be administered to the fracture site to control and optimize the initial inflammatory phase of bone healing. Iloprost is a synthetic analog of prostacyclin (prostaglandin I2; PGI2), approved by the European Medicines Agency (EMA) and the US Food and Drug Administration (FDA) in 2003 and 2004, respectively, for the treatment of primary pulmonary hypertension.” Moreover, the drug has been previously employed as an off-label treatment for bone marrow edema in early cases of osteonecrosis, showing promising therapeutic results [18, 19].

The immunomodulatory and anti-inflammatory effects of systemic iloprost have been previously demonstrated in patients with systemic sclerosis [20]. Recently, in vitro and preclinical animal studies have confirmed the beneficial effect of iloprost as a local immunomodulator in bone regeneration [21]. In an osteotomy model in immune-experienced mice, local iloprost application positively affected bone healing. In this study, iloprost was applied within a fibrin-based delivery system to delay the release of the drug into the osteotomy gap [21]. This delay allows the establishment of the early proinflammatory phase to initiate the healing cascade and provide the necessary microenvironment for the fracture healing process [11, 13].

The local release of iloprost at the fracture site supports the resolution of the initial inflammatory response and promotes the transition to an anti-inflammatory environment by reducing the number of CD8 + T cells and their proinflammatory cytokine secretion [21]. Similarly, iloprost has supported the macrophage switch from the proinflammatory M1 phenotype to the anti-inflammatory M2 phenotype, which is associated with reducing local proinflammatory cytokine levels [22]. In addition, iloprost promoted osteogenic and chondrogenic differentiation of mesenchymal stromal cells in the fracture zone [21] and enhanced local blood flow by improving microcirculation [23]. These results indicate that the local release of iloprost at the fracture site is a suitable approach for enhancing bone fracture healing via the modulation of the inflammatory phase of bone healing [23].

## Methods/design

### Trial design

This study is a pilot phase I/IIa, prospective, mono-center, randomized, open-label, controlled study.

### Participant identification, recruitment, and data collection

Potential participants will be identified from patients admitted to the orthopedic department with Neer type III or IV PHF who are scheduled for open reduction and internal fixation (ORIF) with a proximal humerus internal locking system (PHILOS) (DePuy Synthes, Oberdorf, Switzerland). Patients willing to participate in the study must provide written informed consent after receiving detailed written and oral information concerning the trial. A study physician will enroll the participants. Both the patient and the study physician must sign and date the current version of the study informed consent form. After providing informed consent from an eligible patient, the trial center will formally enroll the participant as a trial patient in the online electronic case report form (eCRF) database. The eCRF software will assign a unique trial identifier to the patient to preserve individual confidentiality. Trial data will be collected and documented soon after measurement by a trained study physician in the eCRF. Captured trial data will be transferred using remote data entry to a central database. The eCRF complies with regulatory standards and good clinical practice (GCP) guidelines and contains the required features, such as an audit, roles and rights management concept, and an electronic signature. Furthermore, the eCRF contains functions to perform plausibility, consistency and range checks of the entered data to obtain high data quality. The quality of the data assembled in the trial database will be checked periodically. Any data inconsistencies will be addressed through queries sent to the trial center for clarification. Every effort will be made to ensure data integrity and minimize missing and defective data throughout the study. All missing or defective data instances will be meticulously documented, and potential causes will be investigated to understand their origin and impact on the study outcomes. At the end of the study, the completed eCRF data set will be closed, investigators’ access will be limited, and the final data set will be provided to the sponsor. The trial subjects will be informed that their data will be stored anonymously to preserve individual confidentiality and used for scientific analysis.

### Dissemination plans

In adherence to the dissemination policy of trial results, the trial results are scheduled for publication in a scientific journal and presentation at different congresses. Any published data will strictly adhere to data protection legislation, ensuring the confidentiality and privacy of the trial subjects and investigators. Additionally, the general outcome of the trial will be available to the study participants.

### Strategies to improve study patient recruitment and retention

Several strategies have been employed to boost patient recruitment. These included developing recruitment materials that are clear, concise, and attractive, effectively explaining the purpose and benefits of the study. Additionally, meetings were held with emergency department physicians, and flyers were distributed to raise awareness of the trial. Trust and credibility were established by providing transparent information about the study, covering the research objectives, methodology, and ethical considerations. Patients have been briefed on the importance of completing the study. Several strategies were implemented to enhance patient retention. Clear communication began early in recruitment and continued throughout the trial. The study has a dedicated phone line that facilitates easy communication with the study team. Regular contact was maintained with participants. They are actively engaged and informed about the trial’s significance and progress. The study visits and procedures were scheduled with some flexibility, accommodating participants’ preferences and schedules whenever feasible.

### Oversight and monitoring

The clinical trial office (CTO Charité) monitors the trial site independently to ensure data quality. Monitoring ensures participant safety, rights, and data accuracy, following GCP principles and local laws. Investigators commit to support regular visits from the monitor, who can compare case report forms with medical records and data protection adherence. Investigators will grant access to all relevant trial documentation for monitoring purposes.

### Data safety monitoring board (DSMB)

A DSMB of independent experts (not directly involved in the conduct of the clinical trial) is convened to monitor the safety and efficacy of the trial and provide impartial advice. The DSMB is composed of three expert members. The DSMB will periodically review the safety data generated, including all adverse events, and recommend whether the protocol should be amended to protect patient safety. Importantly, decisions to terminate the study prematurely based on safety concerns will always involve the DSMB. Further information about roles and meeting frequencies is discussed in the DSMB charter.

### Interim analysis

The clinical study will be subjected to premature termination and interim analysis under any of the following conditions:

- Early evidence of superiority/inferiority for one treatment group.
- Unjustifiable risk or toxicity (decision made by the investigator).
- New scientific evidence provided during the study that demonstrates a risk for patient safety (benefit-risk analysis no longer positive).

### Adverse events evaluation and reporting

Any adverse events encountered by patients participating in the trial will be documented in the eCRF database. These events will be assessed for their severity, seriousness, potential cause, and whether they were expected reactions to iloprost. All adverse events will be addressed appropriately and monitored until resolved or stabilized.

### Trial auditing

Independent trial auditing is being conducted to ensure the quality of trial conduct. Quality control and quality assurance procedures are conducted independently for all trial-related activities. All study procedures strictly follow preestablished standard operating procedures (SOPs) to ensure consistent study quality and adherence to the protocol, the International Conference on Harmonization–Good Clinical Practice (ICH-GCP) guidelines, and relevant regulatory requirements.

### Randomization

Thirty patients will be enrolled in the trial; the study physician will check for eligibility. Eligible patients will be automatically randomized via eCRF computer application on a 1:1:1 basis to one of three groups (group 1: low dose 0.125 ng/kg/min; group 2: high dose 0.25 ng/kg/min; or group 3: the control group, which will be treated with standard of care (SoC) osteosynthesis only). Randomization will be performed using permuted block randomization with stratification by sex to balance sex among the three groups since the incidence of PHFs in females is 2 to 3 times greater than in males [24]. Variable block sizes of 3 and 6 will be used to ensure balanced group sizes without predicting patient allocation and to avoid selection bias.

### Main inclusion criteria

- Patients aged between 40 and 80 years.
- PHF Neer type III or IV scheduled for ORIF with 3-hole PHILOS.
- American Society of Anesthesiologists (ASA) score of ≤ 2.
- Absence of neurovascular complications at the time of trauma.

### Main exclusion criteria

- Immunosuppression due to illness or medication.
- Patients with malignancies undergoing treatment, including chemotherapy, radiotherapy, or immunotherapy.
- Pregnant or breast-feeding women or women of childbearing potential not protected by an effective contraceptive method of birth control (defined as a Pearl index < 1).
- History of previous proximal humerus surgery or deformity on the same side.
- Pathological or open fracture.
- Polytrauma patients.
- Known allergies to iloprost.
- Pulmonary hypertension due to venous occlusive disease.
- Severe coronary heart disease or unstable angina; myocardial infarction within the last six months.
- Acute or chronic congestive heart failure (NYHA II-IV).
- Congenital or acquired valvular defects with clinically relevant myocardial function disorders not related to pulmonary hypertension.
- Patients with any symptomatic or treatable heart disease (including stenting), hypertension treated with a β-receptor blocker, calcium antagonists, vasodilator, or ACE inhibitor at more than moderate doses.
- Conditions where the effects of iloprost on platelets might increase the risk of hemorrhage (e.g., active peptic ulcers or intracranial hemorrhage).
- Patients with a history of cerebral circulatory disorders.

### Intervention

**Fig. 1:**
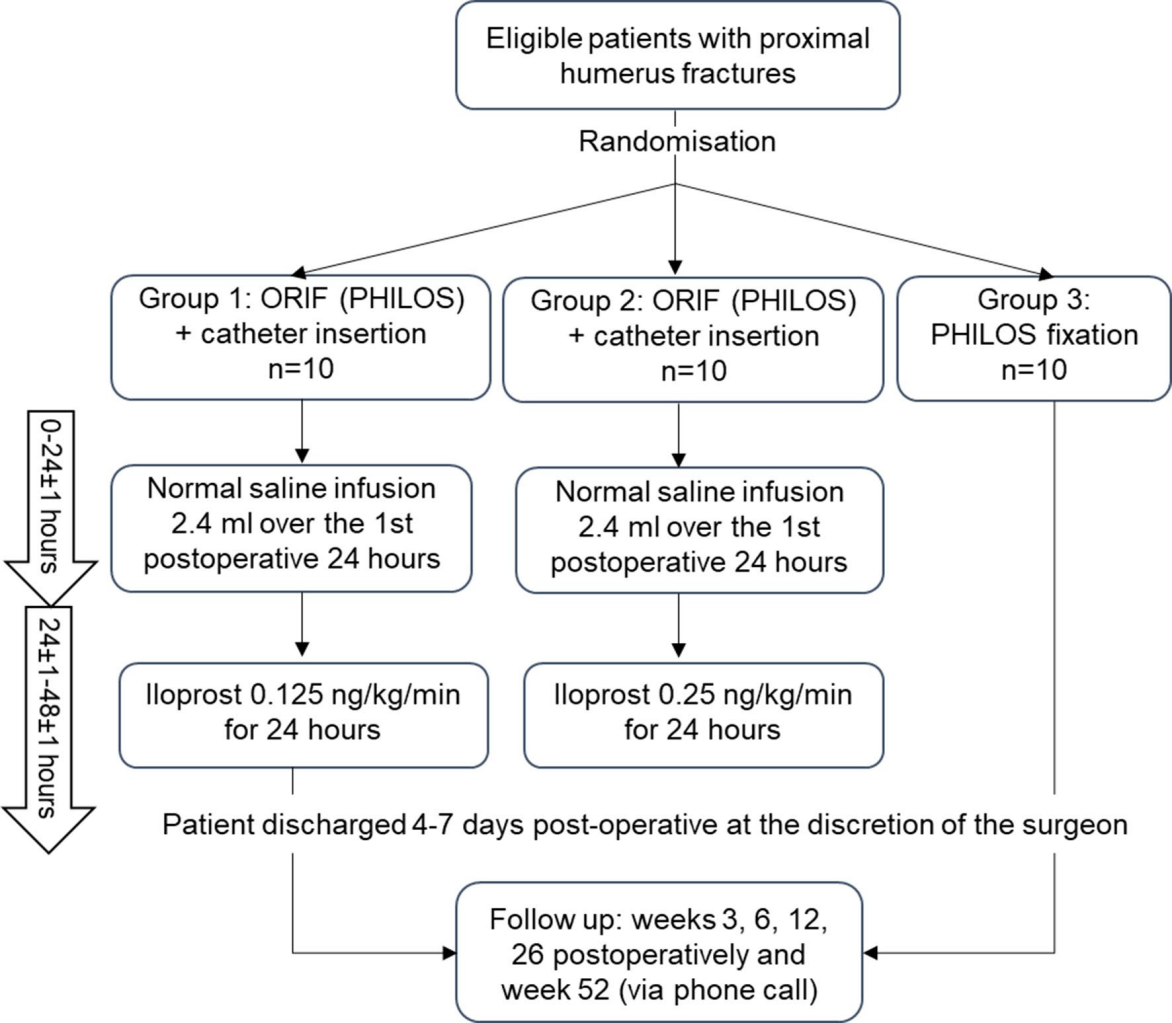
Study flow diagram [26]

All enrolled patients will undergo the SoC operation for PHFs (ORIF with PHILOS), employing a deltopectoral approach. Group 1 (low dose) will additionally receive a total single dose of 0.125 ng/kg/min of iloprost locally administered to the fracture site over 24 h. Group 2 (high dose) will additionally receive a total single dose of 0.25 ng/kg/min of iloprost locally administered to the fracture site over 24 h. The control group (group 3) will only be treated with SoC (ORIF with PHILOS) (Fig. 1). The drug will be applied via a catheter (InfiltraLong 420, PAJUNK^®^ GmbH, Geisingen, Germany), a CE-certified preassembled wound infiltration kit. The catheter will be inserted into the fracture site during the surgical procedure. Both treatment groups will start saline infusion at a low rate (0.1 ml/hour with a total of 2.4 ml/day) during the first 24 h postoperatively to avoid blockage of the catheter; afterward, iloprost treatment will begin for 24 h. The infusion is applied via an infusion pump (CADD solis infusion pump, ICU Medical Inc., California) approved for intraoperative site application with high accuracy (± 6%). At the end of the iloprost application, the catheter is removed by gentle pulling (comparable to a wound drainage system). The prohibited concomitant medications are immunosuppressant agents. All study participants will undergo an identical standard rehabilitation program in accordance with the recommendations of Arbeitsgemeinschaft für Osteosynthesefragen (AO). Patients will be followed up for 52 weeks postsurgery. The study schedule is summarized in Table 1. The study protocol follows the Standard Protocol Items: Recommendations for Intervention Trials 2013 [25]. The primary endpoint assesses the safety and tolerability of a single local iloprost dose applied at the fracture site in PHF patients. The secondary endpoints aim to assess the initial efficacy of the treatment in promoting bone healing in PHF patients. The study endpoints are mentioned in Table 2. Additionally, exploratory biomarker analyses (Table 3) will be conducted to provide deeper insights into the immune modulatory effects of treatment.

The criteria for trial discontinuation include consent withdrawal or, as a decision of the investigator, in case of unjustifiable risk or toxicity to the study patients. The study interventions will not be modified.

**Table 1:**
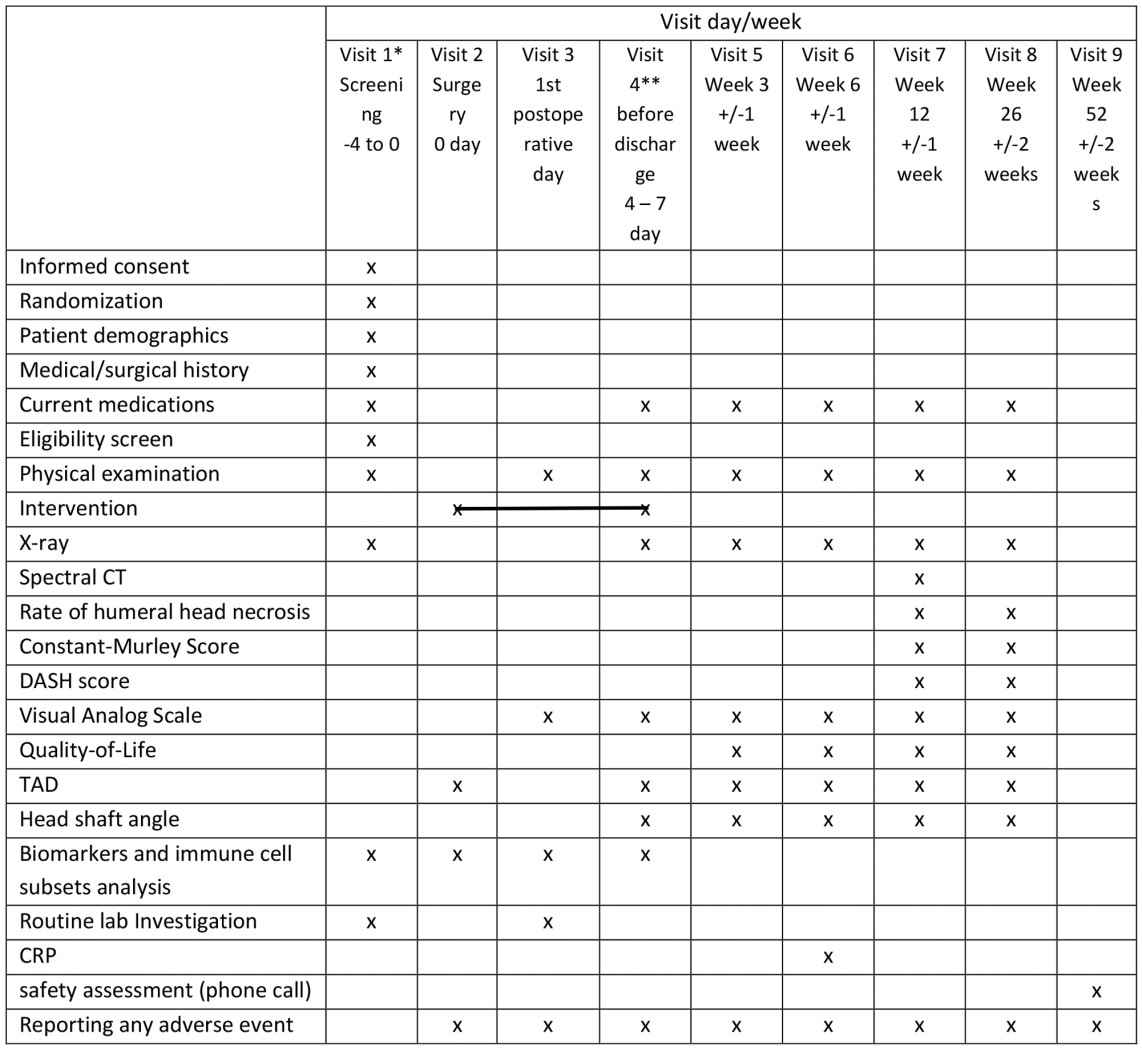
Follow-up schedule. * Recruitment within 24 h of admission, ** postoperative phase before discharging patients from the hospital

### Outcome measures

**Table 2:** Study endpoints

**Primary endpoint** Primary safety and tolerability endpoint: Identification of any noxious response or toxicity that has a causal relationship to the treatment. Toxicity is graded according to the National Cancer Institute Common Terminology Criteria for Adverse Events (NCI-CTCAE). **Secondary endpoints: ** •The degree of preservation of the summation of the tip apex distance for the humeral head screws of the PHILOS at 12 weeks postoperative follow-up visit compared to the postoperative baseline measurement. Tip apex distance refers to the distance between the tip of the screw and the cortex of the humeral head. This radiological endpoint is an indicator of the progress of fracture healing and the probability of potential complications such as loss of reduction [27]. • Rate of humeral head necrosis at the 12- and 26-week visits • Humeral head-shaft angle [28] before discharge, 3, 6, 12, and 26-week visits • Pain assessment using the Visual Analog Scale (VAS) [29] on the first postoperative day, before discharge, 3, 6, 12, and 26-week visits • Quality-of-Life (QoL) by applying EQ-5D [30] at 3, 6, 12, and 26-week visits • Constant-Murley Score (CMS) [31] at 12- and 26-week visits • Disabilities of the Arm, Shoulder, and Hand score (DASH) [32] at 12- and 26-week visits	

### Potential study risk

Risks in this clinical trial can be attributed to the following sources:

### Local administration of iloprost

- Potential adverse events and risk minimization measures.

Clinical evidence from the marketed product Ilomedin^®^ suggests that systemic iloprost infusion is safe and well tolerated. The Ilomedin^®^ data sheet [33] requires a reduction in the administered systemic dose in patients with renal or hepatic impairment. These patients are excluded from the planned study. The study will only include patients with ASA scores of 1 or 2. This refers to either healthy patients or patients with mild-to-well-controlled chronic diseases. Moreover, all contraindications stated in the Ilomedin^®^ data sheet were added to the study’s exclusion criteria. Furthermore, the following precautions will be implemented during the planned trial:

- As recommended in the Ilomedin^®^ data sheet, iloprost will not be initiated in patients with systolic arterial hypotension less than 85 mmHg to avoid further hypotension. Patients will be closely monitored 15 and 30 min after administration, every 2 h during the first 6 h, and then every 6 h until the end of the infusion.
- Care will be taken to avoid potential contamination during the iloprost administration procedure. The procedures will be conducted according to strict standard operating procedures for sterile handling of injection products.
- Because iloprost inhibits platelet function, patients for whom the effects of iloprost on platelets might increase the risk of hemorrhage will be excluded from the study.

b) Local tolerability of iloprost.

Previous preclinical studies performed by our group did not reveal any local toxicity or adverse effects on the cellular composition at or around the fracture gap [21]. In addition to preclinical fracture models, the local application of iloprost has been investigated for various other tissues. For instance, the use of PGI2 analogs such as iloprost and carbaprostacyclin did not result in adverse events in a murine corneal angiogenesis model [34]. The corneal tissue is commonly used to examine the potential angiogenic impact of an experimental drug. The study revealed that iloprost and carbaprostacyclin were able to induce angiogenesis, and more importantly, no signs of local toxicity were noted [34, 35].

The safety and tolerability of local iloprost treatment were also investigated in patients with Peyronie’s disease in a clinical phase I study [36]. Researchers performed intralesional injections of iloprost at a dose of 200 ng in 1 mL of normal saline into penile tissue for five weeks to explore the ability of the drug to suppress the production of connective tissue growth factor in fibroblasts. All patients tolerated the iloprost dose of 200 ng; 19 patients reached a 300 ng dose, and 14 patients tolerated a 400 ng dose without side effects. Only mild side effects (burning or pain) were recorded during the treatment at the site of injection. Overall, the local tolerance of iloprost was of no significant concern in either preclinical or clinical settings [36].

Iloprost is known to have increased side effects at higher infusion rates. In the present study, the application period is four times longer (24 h) than the recommended duration for intravenous iloprost infusion, thereby reducing the risk of side effects.

### Catheter insertion for the delivery of iloprost

Iloprost will be infused locally to the fracture site through an InfiltraLong catheter; the catheter is a CE-certified (No. 51268-16-02) preassembled kit for wound infiltration. The catheter facilitates the diffusion of iloprost into the fracture hematoma (Table 4).

**Table 3:** Exploratory analyses

**Fracture hematoma and blood biomarkers analysis** Trial participants will provide blood and fracture hematoma samples for biomarker analysis, immune cell characterization and further molecular assays in vitro. This analysis could help to identify responders versus nonresponders and gather a more mechanistic understanding of the local immune modulation during fracture healing. Fracture hematoma will be collected as an intraoperative sample, and blood samples will be collected at baseline (visit 1), then 24, 48, and 96 h after surgery. **Spectral computed tomography (sCT)** Shoulder spectral computed tomography (sCT) for the operated side will be performed at the 12-week study visit. The sCT scan will ensure a better assessment of bone healing and serve as a reliable prognostic factor for potential complications such as loss of reduction and nonunion [37, 38].	

**Table 4:** Administrative information

Trial title	ILOBONE: A phase I/IIa randomized controlled trial to assess the safety and feasibility of local iloprost therapy for enhancing proximal humerus fracture healing– a pilot study design
Trial registration	ClinicalTrial.gov (NCT04543682) registered 02 Sep. 2020, https://clinicaltrials.gov/show/NCT04543682 and the German Clinical Trials Registry (DRKS00027081), registered 10 Nov. 2021 https://drks.de/search/de/trial/DRKS00027081
Protocol version	Version 6, 30 Sep. 2022.
Trial status	The study is recruiting; the recruitment began on 21.09.2022. The recruitment is expected to be completed in the second quarter of 2025.
Declaration of interests	The study is not co-financed. The independence of investigators is ensured.
Roles and responsibilities: sponsor contact information	Investigator-initiated trial Charité– Universitätsmedizin Berlin Center for Musculoskeletal Surgery Julius Wolff Institute and Berlin-Brandenburg Center for Regenerative Therapies (BCRT) Augustenburger Platz 1, 13,353 Berlin Germany Sponsor representative: Prof. Dr. Med. Tobias Winkler

### Provisions for post‑trial care

If needed, patient follow-up after the trial period is ensured by the Charité Center for Musculoskeletal Surgery. According to regulatory requirements, specific insurance is provided to compensate individuals who might suffer harm as a result of their participation in the clinical trial.

### Statistical analysis

Primary Safety Analysis: Safety endpoints will be evaluated descriptively, with all adverse events summarized for each patient. Serious adverse events will be further evaluated according to frequency, severity, and treatment-relatedness. Potential risk factors for serious adverse events, including patient demographics and medical history, will be investigated by analyzing the safety endpoint in relation to various patient characteristics.

Efficacy Analysis: Descriptive statistics will be performed according to the different data types (frequencies, mean ± standard deviation, median and range) for summarizing the preliminary efficacy endpoints. Furthermore, we will report estimated group differences along with 95% simultaneous confidence intervals obtained from a Dunnett-type (many-to-one) or Tukey-type (many-to-many) contrast test, allowing for variance heteroscedasticity. Since sample sizes are rather small, critical values and p-values will be obtained from a multivariate t-distribution with Satterthwaite-type degrees of freedom.

The primary analyses will follow an intention-to-treat approach, using all available data. Missing values will not be imputed, and subsequent analyses addressing missing data will be considered exploratory.

## Discussion

The primary trial objective is to establish the safety, feasibility and tolerability of a local iloprost single-dose application at the fracture site in patients with PHFs. The secondary objective is to assess the preliminary efficacy of the treatment on bone healing. The study design is a randomized controlled trial utilizing quantitative and qualitative data collection methods to assess the safety and preliminary efficacy of iloprost for bone healing. The data obtained from the Ilobone study will provide crucial information on effect size estimates, which are essential for calculating the sample size for a future, larger-scale clinical trial focused on efficacy.

Conclusions: The Ilobone study aims to provide data on the potential for biological augmentation of osteosynthesis procedures in a type of fracture, the PHF, which is prone to healing challenges and complications.

## Electronic supplementary material

Below is the link to the electronic supplementary material.

Supplementary Material 1

## Data Availability

No datasets were generated or analysed during the current study.

## Abbreviations

ACE: Angiotensin-converting enzyme
ASA: American Society of Anesthesiologists
CMS: Constant-Murley score
DASH: Disabilities of the Arm, Shoulder, and Hand Scale
DSMB: Data Safety Monitoring Board
Ecrf: Electronic case report form
GCP: Good clinical practice
ICH: GCP-The International Conference on Harmonization-Good Clinical Practice
IFNɣ: Interferon-gamma
NYHA: New York Heart Association Classification
ORIF: Open reduction and internal fixation
PHFs: Proximal humerus fractures
PHILOS: Proximal humerus internal locking system
QoL: Quality of Life
SoC: Standard of care
SOPs: Standard operating procedures
TNFα: Tumor necrosis factor-alpha
VAS: Visual Analog Scale
