# Supplementary material for: ILOBONE: A phase I/IIa randomized controlled trial to assess the safety and feasibility of local iloprost therapy for enhancing proximal humerus fracture healing– a pilot study design
Source: J Orthop Surg Res. 2025 May 22;20:498. doi: 10.1186/s13018-025-05865-2 (PMC12096472; doi:10.1186/s13018-025-05865-2)
Supplement: Supplementary file 1 — Supplementary Material 1 [file 13018_2025_5865_MOESM1_ESM.pptx]

## Slide 1
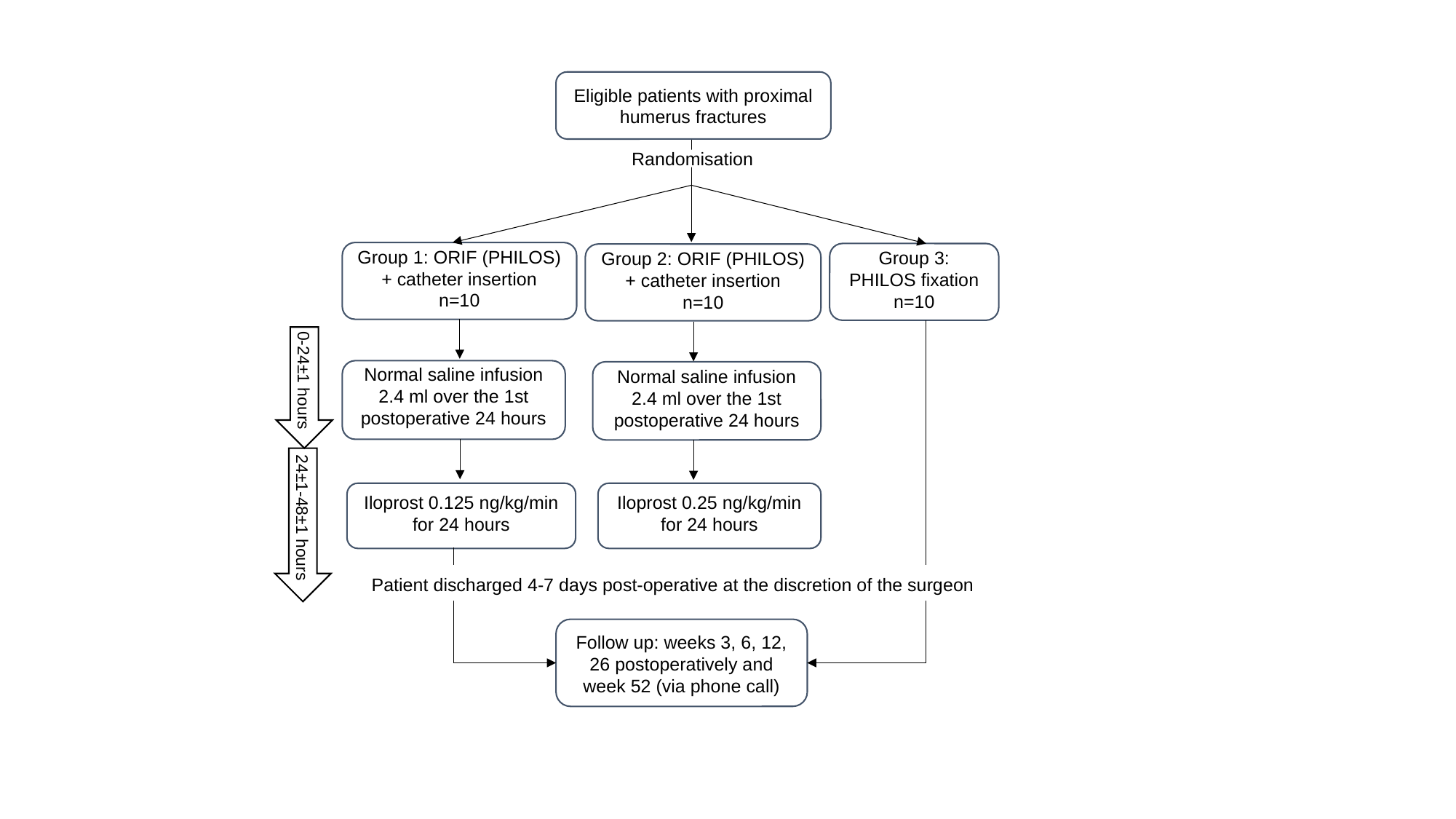

Eligible patients with proximal humerus fractures
Randomisation
Group 1: ORIF (PHILOS) + catheter insertion
n=10
Group 3: PHILOS fixation
n=10
Group 2: ORIF (PHILOS) + catheter insertion
n=10
0-24±1 hours
Normal saline infusion 2.4 ml over the 1st postoperative 24 hours
Normal saline infusion 2.4 ml over the 1st postoperative 24 hours
Iloprost 0.125 ng/kg/min for 24 hours
Iloprost 0.25 ng/kg/min for 24 hours
24±1-48±1 hours
Patient discharged 4-7 days post-operative at the discretion of the surgeon
Follow up: weeks 3, 6, 12, 26 postoperatively and week 52 (via phone call)
